# Chemical Characterization and Migration Profile of Silicone Molds for Air Fryers

**DOI:** 10.3390/polym18172162

**Published:** 2026-09-04

**Authors:** Janira Jaén, Cristhopher Castillo, Lourdes Arjona, Paula Vera, Elena Canellas, Cristina Nerín, Jorge González-Chan, Felipe Barria-Almengor, Gerardo Cáceres, Carlos H. Ríos Martínez

**Affiliations:** 1 Analytical Chemistry Department, Universidad de Panamá, Simón Bolivar Avenue, P.O. Box 3366, Panama 0824, Panama; cristhopher.castillo@up.ac.pa (C.C.); jorge.gonzalezc@up.ac.pa (J.G.-C.); felipe.barria@up.ac.pa (F.B.-A.); 2 Sistema Nacional de Investigadores (SNI), SENACYT, Panama 0801, Panama; 3 Organic Chemistry Department, Universidad de Panamá, Simón Bolivar Avenue, P.O. Box 3366, Panama 0824, Panama; lourdes.arjona@up.ac.pa (L.A.); gerardo.caceres@up.ac.pa (G.C.); carlos.rios-m@up.ac.pa (C.H.R.M.); 4 GUIA Group, Analytical Chemistry Department, I3A, EINA, University of Zaragoza, María de Luna 3, 50018 Zaragoza, Spain; pvera@unizar.es (P.V.); elenac@unizar.es (E.C.); cnerin@unizar.es (C.N.)

**Keywords:** silicone elastomers, migration, silicone molds, air fryers, NIAS, siloxanes, repeated use, food contact materials

## Abstract

The chemical profile and migration behavior of volatile and semi-volatile compounds from silicone molds intended for use in air fryers were investigated. The study combined migration tests using 10% ethanol, 3% acetic acid, and simulant E (Tenax^®^) with direct headspace (HS) analysis of the molds by gas chromatography–mass spectrometry (GC-MS). HS analysis and migration tests with aqueous simulants were performed over three heating cycles to simulate repeated use. A non-targeted analytical approach was applied, followed by selective quantification based on toxicological criteria and the relevance of the compounds to the material. A total of 128 substances were identified, including siloxanes and non-intentionally added substances (NIASs). Siloxanes were detected in both simulant E and 10% ethanol. Most NIASs were found at levels below the limit of quantification or within regulatory limits, except for dibutyl phthalate, which exceeded its specific migration limit (0.3 mg kg^−1^). Furthermore, the sum of migrated cyclic siloxanes in simulant E surpassed the generic specific migration limit (60 mg kg^−1^) established in Spanish Royal Decree 847/2011, while the release of volatile organic compounds exceeded the recommended value of 0.5%. The results revealed a complex chemical profile characterized by diverse NIASs and elevated migration of cyclic siloxanes in simulant E.

## 1. Introduction

Silicone elastomers are commonly used in food-contact materials due to their flexibility, thermal stability, chemical resistance, and non-stick properties [1,2]. Among their applications are molds designed for air fryers, whose use has increased with the growing adoption of these devices for food preparation at elevated temperatures.

Silicone elastomers are siloxane-based polymers whose backbone consists of silicon–oxygen (Si–O–Si) linkages, with organic groups attached to the silicon atoms [2,3]. They are synthesized via the hydrolysis of chlorosilanes to form silanols (R_3_SiOH), followed by condensation to form siloxane bonds (Si–O–Si) with the release of water [4]. This structure imparts high thermal stability and hydrophobicity to these materials [3,5].

Subsequently, siloxane polymers undergo crosslinking and curing, which confer elastomeric properties [6]. During these processes, low-molecular-weight oligomers may remain in the material and be released upon heating [7]. The German Federal Institute for Risk Assessment (BfR) recommends that silicone materials intended for food contact undergo thermal post-curing after manufacturing to ensure that the total release of volatile organic compounds does not exceed 0.5% (*w*/*w*) [8,9]. The implementation of this practice may vary depending on the manufacturer and the target market.

In recent years, several studies have reported the migration of siloxanes from silicone materials into food at high temperatures, as well as the release of non-intentionally added substances (NIASs), including plasticizers, lubricants, and metals [10,11,12,13]. Most of these studies have focused on the migration of specific cyclosiloxanes under single-contact conditions, whereas the migration of NIASs under repeated-use conditions has received less attention.

Recently, Wrona et al. evaluated 44 silicone molds marketed in Europe using aqueous simulants and repeated-use tests, providing valuable information on migration behavior [14]. However, given the compositional variability among commercial products, there remains a need to generate data from other markets to further expand the understanding of migration profiles across different scenarios.

In addition, cyclic siloxanes, such as octamethylcyclotetrasiloxane (D4), decamethylcyclopentasiloxane (D5), and dodecamethylcyclohexasiloxane (D6), have been linked to impaired fertility, endocrine-disrupting effects, and bioaccumulation [15,16,17,18]. These compounds have been identified by the European Chemicals Agency (ECHA) as substances of very high concern due to persistent and bioaccumulative properties [19]. This reinforces the interest in evaluating migration from silicone molds, particularly in emerging markets, where limited technical information on labeling and the lack of specific controls may increase uncertainty about safety.

Currently, there is no harmonized legislation specifically regulating migration testing for silicone food-contact materials. Consequently, migration studies commonly apply the testing protocols established in Regulation (EU) No. 10/2011 for plastic materials [20]. In line with this approach, the present research adopts this regulatory framework as a reference for experimental design and safety assessment.

This work aims to characterize the chemical profile and migration behavior of compounds released from silicone molds used in air fryers through a combination of headspace (HS) analysis, migration tests, and untargeted gas chromatography–mass spectrometry (GC-MS). Furthermore, the application of this methodology enables the identification and quantification of compounds that are potentially relevant from analytical and regulatory perspectives.

## 2. Materials and Methods

### 2.1. Reagents and Materials

Octamethylcyclotetrasiloxane (556-67-2), decamethyltetrasiloxane (141-62-8), decamethylcyclopentasiloxane (541-02-6), dodecamethylcyclohexasiloxane (540-97-6), benzophenone (119-61-9), naphthalene (91-20-3), dimethyl phthalate (131-11-3), diisobutyl phthalate (84-69-5), diphenyl sulfone (127-63-9), dibutyl phthalate (84-74-2), diphenyl ether (101-84-8), 2,4-di-tert-butylphenol (96-76-4), 2,2,4-trimethyl-1,3-pentanediol diisobutyrate (6846-50-0), 2,6-di-tert-butyl-1,4-benzoquinone (719-22-2), hexadecanoic acid (57-10-3), dodecan-1-ol (112-53-8), and methyl oleate (112-62-9), together with a mixture of n-alkanes (C10–C50) in n-hexane (approximately 1000 µg g^−1^), were purchased from Sigma-Aldrich (Madrid, Spain).

Tenax^®^ TA (60/100 mesh, poly(2,6-diphenyl-p-phenylene oxide)) and the DVB/CAR/PDMS fiber (divinylbenzene/carboxen/polydimethylsiloxane) were supplied by Supelco (Bellefonte, PA, USA). Analytical-grade solvents, including absolute ethanol, acetone, and acetic acid, were purchased from Scharlab S.L. (Barcelona, Spain). Type I ultrapure water was obtained using an Ultramatic GR purification system (Wasserlab, Barbatáin, Spain).

### 2.2. Sample

Ten silicone molds marketed in Panama for use in air fryers were evaluated. Sample characteristics, including identification code, color, and manufacturer-provided information, are summarized in Table 1, and Figure S1 shows some of the different silicone molds evaluated in this study.

### 2.3. Gravimetric Determination of Total Volatile Organic Compounds

The release of volatile organic compounds (VOCs) was evaluated using a gravimetric method, following the procedure described by Oliveira et al. [22], with slight modifications. Samples were cut into fragments of approximately 1 × 1 cm and conditioned for 87 h in a desiccator containing previously regenerated silica gel with a moisture indicator.

Subsequently, 2 g of each sample was weighed into glass vials and subjected to thermal treatment at 200 °C for 4 h. After heating, the vials were placed again in the desiccator for 1 h to reach room temperature and prevent moisture uptake. The final mass was then recorded. The experiment was performed in triplicate for each sample, and the mass loss was used to calculate the percentage of VOCs released.

### 2.4. Headspace Analysis Under Thermal Cycling

For this study, samples were cut into small fragments of approximately 0.5 cm and placed into 20 mL glass vials, which were hermetically sealed and heated in an oven at 100 °C for 4 h to simulate repeated-use conditions. Each sample was subjected to three consecutive heating cycles.

HS analysis was conducted after the first and third cycles to assess the initial release of volatile compounds and changes upon successive exposures. The analyses were carried out using headspace solid-phase microextraction coupled with gas chromatography–mass spectrometry (HS-SPME-GC-MS). The experiment was performed in triplicate for each sample.

### 2.5. Migration Tests

Migration tests were carried out under standardized test conditions selected according to Regulation (EU) No. 10/2011 on plastic materials intended to come into contact with food and following the recommendations of the Joint Research Centre (JRC) Technical Report on silicone and rubber materials [20,23].

For the experiments, 10% ethanol (simulant A), corresponding to foods of a hydrophilic character; 3% acetic acid (simulant B) for acidic foods; and Tenax^®^ (simulant E) for dry or low-moisture matrices were selected. These simulants were used to evaluate migration under contact conditions with different physicochemical characteristics and to assess the influence of the contacting medium on the migration profile. Foods with a high fat content were not specifically represented by the selected simulants; therefore, the present study does not cover all possible food-contact conditions associated with the use of silicone molds in air fryers. All tests were performed in triplicate and included duplicate blank samples, which were subjected to the same procedure as the test samples.

#### 2.5.1. Aqueous Simulants

In migration tests using aqueous simulants, due to the size and open geometry of the molds, representative samples were cut into rectangular pieces with a total exposed surface area of 10.8 cm^2^, maintaining the surface area-to-volume ratio of 6 dm^2^ kg^−1^ established in Regulation (EU) No. 10/2011 (see Figure S2). Each sample was placed in a glass vial, to which 18.0 mL of simulant was added, with the volume gravimetrically controlled to ensure exact volume and complete immersion of the sample.

Three consecutive migration tests were performed on the same material at 100 °C for 4 h. Migration was evaluated in the first and third tests to examine the initial profile and its variation after successive contacts. Analysis was carried out using SPME-GC-MS, with direct immersion of the fiber in the simulant.

#### 2.5.2. Solid Simulant

Before the test, Tenax^®^ was purified by Soxhlet extraction with acetone for 6 h and dried in an oven at 160 °C, following the procedure described in UNE-EN 14338 [24]. During the experiment, 0.30 g of Tenax^®^ was placed in contact with the silicone sample, using the 4 g dm^−2^ ratio established in the same standard. Tenax^®^ was applied exclusively to the original food-contact surface of the silicone specimen, using an aluminum support designed to confine the simulant to that surface and prevent contact with the cut edges. The silicone–Tenax^®^ assembly was carefully wrapped in aluminum foil, placed in a glass Petri dish, and subjected to thermal treatment at 175 °C for 1 h.

After the experiment, the migration analytes retained in the Tenax^®^ were extracted by three consecutive extractions with 4 mL of ethanol using an ultrasonic bath at room temperature for 1 h each. The extracts were combined in a clean vial and centrifuged to remove sorbent residues. The supernatant was concentrated to a final volume of 2 mL at 40 °C under a nitrogen stream and analyzed by GC-MS.

### 2.6. Instrumental Conditions

Analyses were performed using an Agilent 8860N gas chromatograph coupled to an Agilent 5977B mass spectrometer and equipped with an HP-5 capillary column (30 m × 250 µm i.d. × 0.25 µm film thickness), all from Agilent Technologies (Santa Clara, CA, USA). A CTC Analytics PAL RSI 85 autosampler (CTC Analytics AG, Zwingen, Switzerland) was used. The injector temperature was set at 250 °C. Injections were performed in splitless mode using helium as the carrier gas. The flow rate was maintained at 1 mL min^−1^.

The oven temperature program started at 50 °C and was held for 5 min, then ramped at 10 °C min^−1^ to 300 °C, where it was held for 5 min. The mass spectrometer operated in SCAN mode over a mass range of 45–450 *m*/*z*, using electron ionization (EI) at 70 eV, with a solvent delay of 5 min and an ion source temperature of 250 °C.

Volatile compounds in the HS and in aqueous simulants were analyzed using a DVB/CAR/PDMS SPME fiber (50/30 µm). Extraction conditions, previously optimized, included incubation at 80 °C for 5 min, an extraction time of 20 min, and desorption for 5 min. For compounds migrated into Tenax^®^, 1 µL of the extract was injected under the same chromatographic conditions described above.

### 2.7. Compound Identification

The first step in the identification process consisted of comparing the chromatograms of the samples with their corresponding blanks, analyzed using the same analytical procedures. Substances absent in the blanks or those whose peaks showed significantly higher intensity were considered of interest.

Identification was carried out by comparing mass spectra with the NIST reference library (version 17), considering match factors ≥ 800. This was complemented with experimental retention indices (RI_e_), calculated from a mixture of n-alkanes (C7–C40) analyzed under the same chromatographic program and compared with those reported in specialized databases (RI_l_), including the NIST Chemistry WebBook (SRD 69), The Pherobase, and FlavorNet. When possible, the identity of the substances was confirmed by injecting analytical standards and comparing the retention times and mass spectra obtained with those of the reference.

The detected siloxanes were classified as cyclic, linear, or derivatives according to chemical structure, fragmentation patterns, and chromatographic behavior. In this study, siloxane species whose spectral information did not allow for individual structural assignment were designated as derivatives. Consequently, these signals were operationally classified as Dn or Ln derivatives, based on the predominant fragmentation pattern for identification purposes.

### 2.8. Selection and Quantification of Compounds of Interest with Toxicological Relevance

For the selection of analytes of interest, inclusion in the positive list of Regulation (EU) No. 10/2011 was first verified to identify authorized substances and the existence of specific migration limits (SMLs). Subsequently, the compounds were classified according to chemical structure into the three categories of the Cramer classification scheme: Class I (low toxicological risk), Class II (intermediate risk), and Class III (high potential risk), using Toxtree v3.1.0 software.

Based on this assessment, substances with regulatory or toxicological relevance were selected for quantification. These included compounds with SMLs, those classified as Cramer Class III, siloxanes, and other silicon-containing species, given the direct relationship to the material. Phthalates were also considered due to their relevance in risk assessment for food-contact materials.

Quantification was performed using external calibration curves prepared in 10% ethanol, 3% acetic acid, and absolute ethanol, verifying linearity across the studied concentration range. Limits of detection (LOD) and quantification (LOQ) were estimated from the signal-to-noise ratio (S/N), using the lowest detected analyte levels and S/N thresholds of 3 for LOD and 10 for LOQ, respectively. When an analytical standard was unavailable, a semi-quantitative approach was applied using compounds with similar chemical structures (see Tables S1 and S2).

Preliminary risk assessment was carried out by comparison with the SMLs established in Regulation (EU) No. 10/2011. For quantified substances without an established SML and classified as Cramer Class III, the threshold of toxicological concern (TTC) approach was applied using the Cramer Class III threshold of 90 µg/person/day (equivalent to 90 ng g^−1^ of food). For siloxanes, given the limitations of the Cramer scheme for this type of substance, the assessment was based on the generic specific migration limit of 60 mg kg^−1^ of food (6.0 × 10^4^ ng g^−1^) established in Spanish Royal Decree 847/2011 [25]. This comparison does not constitute an overall migration test.

## 3. Results and Discussion

### 3.1. Release of Total Volatile Organic Compounds

**Figure 1:**
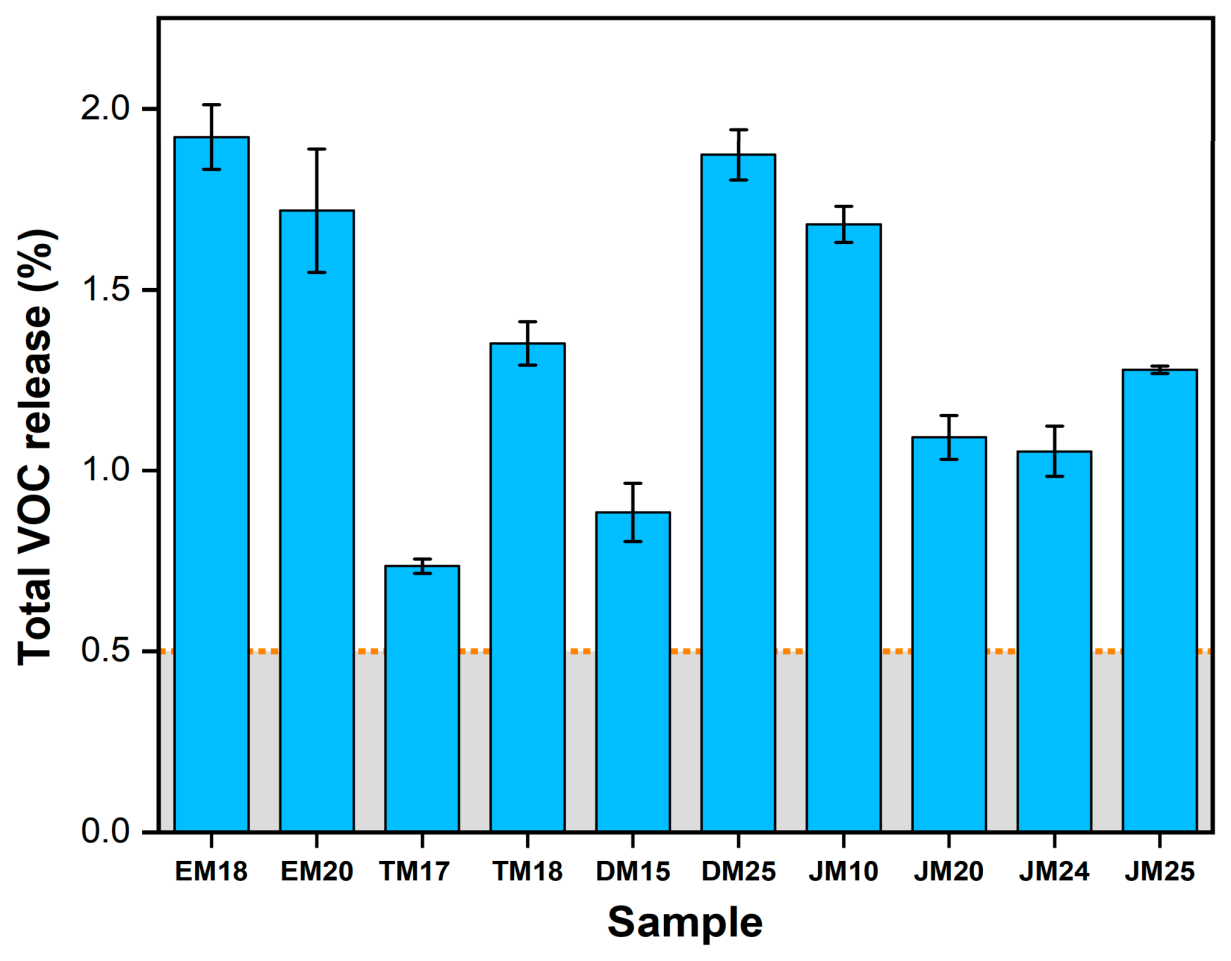
Total volatile organic compound (VOC) release from silicone molds and comparison with the BfR recommended threshold (0.5%). The orange dashed line indicates the threshold, while the grey shaded area represents values below this limit.

Figure 1 shows the total percentage of VOCs released by the analyzed silicone molds. All samples exceeded the reference threshold of 0.5% (*w*/*w*) recommended by the BfR for silicone materials intended for food contact. Samples EM18 and DM25 showed the highest release percentages, approaching 2%, whereas samples TM17 and DM15 showed the lowest values, although still above the recommended limit. These results suggest differences in the formulation and curing conditions of the silicone materials.

This behavior can be interpreted in relation to the formation and subsequent thermal conditioning of the silicone elastomer network. During curing, polysiloxane chains are crosslinked to form a three-dimensional elastomeric network. However, not all species present during silicone manufacturing are incorporated into this network. Therefore, a mobile fraction consisting of cyclic and linear siloxane oligomers may remain physically trapped within the polymer matrix. The amount and composition of this fraction depend on the silicone formulation, crosslinking system, and curing conditions [26,27,28,29].

Post-curing plays an important role in reducing volatile compounds and residual oligomers in silicone elastomers by promoting their removal through diffusion and evaporation during thermal treatment [28]. Consequently, insufficient post-curing may result in a larger fraction of mobile species that can be released during subsequent heating [12,29,30].

The high VOC levels observed in all samples are consistent with the presence of an appreciable mobile fraction and may indicate insufficient thermal conditioning after manufacturing. Therefore, it has been recommended that silicone molds undergo heat treatment prior to contact with food to reduce exposure to volatile substances released from the material [12]. This is particularly relevant when labeling does not provide detailed information on manufacturing processes or clearly indicate whether the material meets food-grade criteria, as observed in emerging markets such as Panama [31].

### 3.2. Compound Identification

In total, 128 compounds were detected in migration assays and headspace (HS) analysis. These are listed in Table 2 along with their retention time (RT), CAS number, molecular formula, the media in which they were detected (HS, simulants A, B, and E), and the corresponding cycle (1 or 3). Additionally, the experimental retention index (RI_e_) and the literature retention index (RI_l_), the detection frequency (DF), the classification according to the Cramer scheme, and the available SML information are included.

As shown, the identified VOCs, in addition to organosilicon species (cyclic and linear siloxanes), include other substances of diverse chemical nature. This profile reflects the complexity of silicone materials and can be attributed to factors such as elastomer formulation, manufacturing and curing processes, thermal degradation, and the presence of substances of environmental origin.

#### 3.2.1. Headspace

In the HS analysis, 40 compounds were detected, 29 of which corresponded to non-siloxane species. Most were identified during the first heating cycle, whereas some medium-chain aliphatic ketones (2-heptanone, 2-octanone, 2-nonanone, and 2-decanone) were observed only in the third cycle, suggesting delayed release or heat-induced formation.

Some of these analytes may be related to the ability of silicone materials to absorb VOCs from the environment during storage, transport, or distribution. Silicone elastomers exhibit high affinity for hydrophobic species and high gas permeability, promoting environmental sorption of VOCs [32,33]. Once incorporated into the material, these compounds may be released upon exposure to elevated temperatures. This external contribution is consistent with the occurrence of substances not directly associated with the structure of the silicone matrix.

In the case of siloxanes, 10 cyclic and 1 linear species were identified in both the first and third cycles. This behavior indicates that these are not only surface residues, but rather volatile substances retained within the silicone network. The predominance of cyclic structures is consistent with higher volatility and molecular mobility, explaining the presence in the HS.

Furthermore, 24 components were found exclusively in the HS and not in the food simulants, highlighting the complementarity of the two analytical approaches. Taken together, these results provide a more comprehensive characterization of the compounds released from the silicone molds.

#### 3.2.2. Simulants

As shown in Table 2, a total of 104 substances were identified in the migration tests. In simulant A, 60 were detected in the first migration and 34 in the third, while in B, 40 were observed in the first and 30 in the third. In simulant E, 59 species were identified.

Migration showed a clear dependence on the nature of the medium. Simulant A exhibited a greater capacity to mobilize substances from the matrix compared with B, which can be attributed to the presence of ethanol, as it reduces the polarity of the medium and favors interaction with nonpolar molecules [34]. In both aqueous simulants, the third migration was characterized by a decrease in the number of initially identified species and the appearance of new substances, indicating compositional changes after successive contacts. Siloxanes migrated predominantly to the solid simulant, followed by simulant A, while no migration was observed in simulant B. Cyclic siloxanes up to D9 were detected in both simulant A and Tenax, whereas larger siloxanes, such as D10 and its derivatives, were found only in simulant E. Linear siloxanes were present in smaller numbers and mainly in the latter.

On the other hand, among the non-siloxane migrants, butylated hydroxytoluene (BHT), previously reported in silicone molds and teats [12,35], was detected. Degradation products of phenolic antioxidants were also observed, along with plasticizing esters, including phthalates (dibutyl phthalate DBP, dimethyl phthalate DMP, diisobutyl phthalate DIBP, diethyl phthalate DEP), and other additives such as 2,2,4-trimethyl-1,3-pentanediol diisobutyrate.

Aromatic ketones, such as benzophenone and related compounds, including (4-methylphenyl)phenylmethanone and (4-chlorophenyl)phenylmethanone, described as UV photoinitiators in polymeric formulations [36,37], were identified. In addition, aliphatic ketones, aromatic compounds, and medium- and long-chain fatty acids were detected; this is consistent with the use of processing aids and secondary additives employed during polymer manufacturing, as well as with heating-induced side reactions [1,38].

Substances characteristic of fragrances or cosmetic formulations, such as α-terpineol, cedrol, and isobornyl acetate, were also recorded and may be related to the absorption of environmental VOCs. In addition, two functionalized organosilicate compounds of the orthosilicate type were identified, structurally distinct from cyclic and linear siloxane oligomers, whose presence may be due to residues from synthesis or byproducts of the material manufacturing process.

### 3.3. Quantification of Selected Compounds and Potential Risk Assessment

The analytes selected based on the non-targeted analysis were quantified in the different simulants using external calibration. These data were used for a preliminary risk assessment based on regulatory and toxicological criteria. Due to differences in concentration ranges, results are expressed in ng g^−1^ in aqueous matrices and in mg kg^−1^ for Tenax. The analytical parameters for both methods (working range, linearity, LOD, and LOQ) and the calibration standards are presented in Tables S1 and S2 of the Supplementary Materials.

#### 3.3.1. Non-Siloxane Compounds

The concentrations of the non-siloxane compounds are presented in Tables S3–S5. Most were found below the method LOQ (see Table S1). However, some analytes were detected at quantifiable levels. In the aqueous simulants, these included hexadecanamide (43.0 ± 8.5 ng g^−1^), (Z)-octadec-9-enamide (53.1 ± 2.8 ng g^−1^), and (4-chlorophenyl)phenylmethanone (16.1 ± 3.5 ng g^−1^) in the first migration of simulant A, as well as diphenyl sulfone (30.4 ± 3.7 ng g^−1^) in the third migration of simulant B. In Tenax, functionalized organosilicate compounds of the orthosilicate type were quantified at concentrations of up to 1.1 × 10^3^ ng g^−1^.

Among the compounds mentioned above, only diphenyl sulfone is included in the positive list of Commission Regulation (EU) No 10/2011, with an SML of 3.0 mg kg^−1^ of food, which was not exceeded. The other compounds were detected at concentrations below the generic specific migration limit established for non-listed substances. In addition, compounds assigned to Cramer Class III remained below the threshold considered for substances of potential toxicological concern.

**Figure 2:**
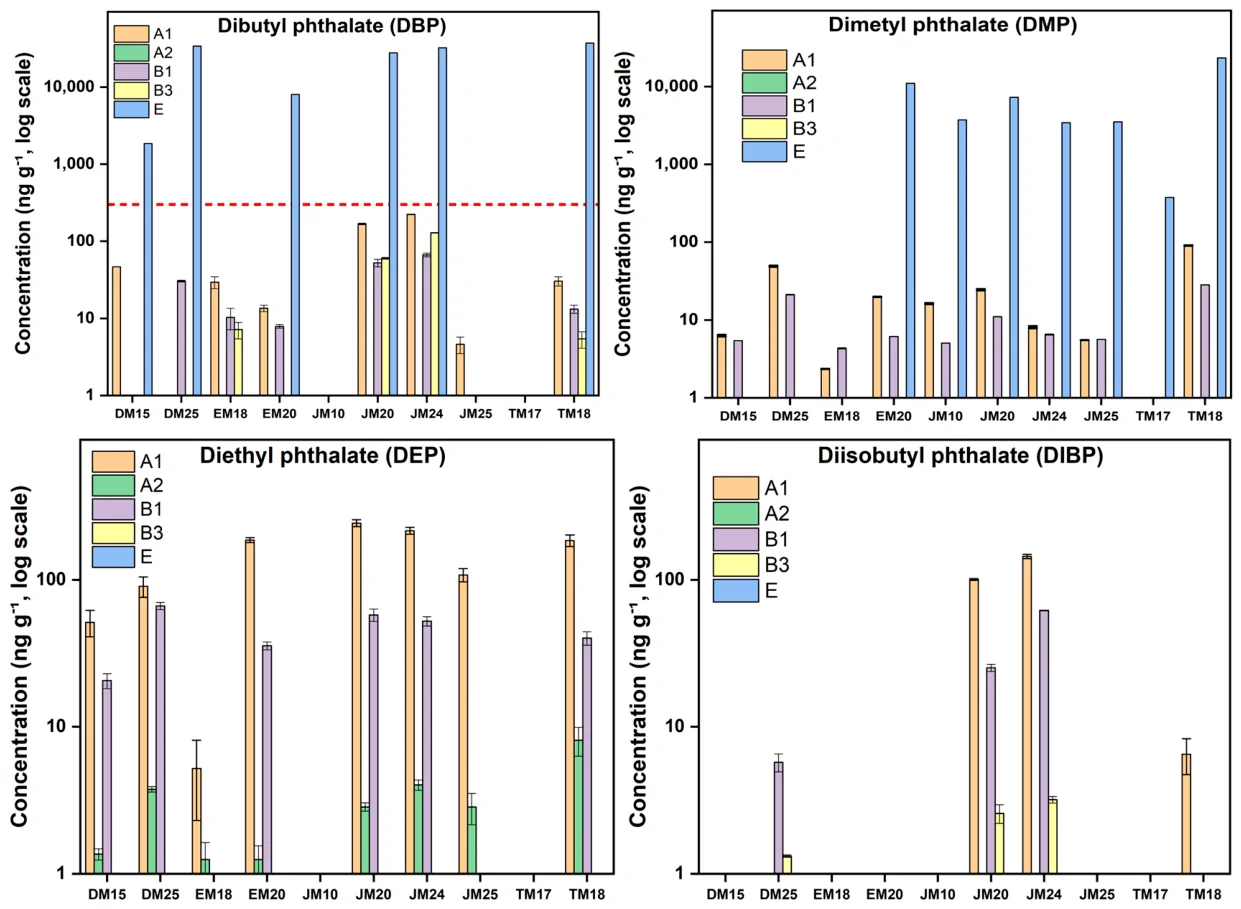
Migration of phthalates (DBP, DMP, DIBP, and DEP) in simulants A, B, and E. Concentrations are expressed as ng g^−1^ on a logarithmic scale. The red line indicates the specific migration limit (SML) for DBP (300 ng g^−1^).

On the other hand, Figure 2 shows phthalate migration. In aqueous simulants, DEP and DBP exhibited the highest values, reaching 243.1 and 223.7 ng g^−1^, respectively, followed by DIBP at 144.4 ng g^−1^, whereas DMP remained below 91 ng g^−1^. In Tenax, only DMP and DBP were detected; however, migration occurred at markedly higher levels, on the order of 10^4^ ng g^−1^.

Phthalates showed different behaviors during repeated use. DMP and DEP decreased from the first to the third migration in both aqueous simulants, whereas DBP and DIBP showed greater variability. In particular, DBP decreased in simulant A, but in simulant B it remained quantifiable after the third migration and, in some samples, reached concentrations higher than those observed in the first migration. These differences should be considered taking into account that the molds studied are commercial products from different manufacturers and that phthalates are not chemically bound to the silicone matrix; therefore, their presence and concentrations may vary among samples. Furthermore, DMP, DEP, DIBP, and DBP have different physicochemical properties and PDMS/water partition coefficients, factors that may influence their migration behavior [39].

Due to their toxicological relevance, several phthalates are subject to specific migration limits and group restrictions associated with reproductive toxicity and potential endocrine-disrupting effects [40,41]. Among the phthalates identified, only DBP has an assigned SML of 0.3 mg kg^−1^ of food. In the solid simulant, this analyte reached concentrations of up to 3.7 × 10^4^ ng g^−1^, whereas values detected in the aqueous simulants remained below the limits established by European regulations.

#### 3.3.2. Siloxane Compounds

Quantitative analysis revealed substantial differences between simulants A and E. In simulant A, siloxane concentrations remained below the method’s LOQ (Tables S1 and S2), whereas elevated levels were quantified in simulant E (Table S5), comparable to those reported for low-quality silicone molds [12].

Given the variability in siloxanes migrating into simulant E, box-and-whisker plots were used to characterize the distribution of different siloxane types by visualizing central tendency, dispersion, extreme values, and relative contributions to the quantified siloxane profile.

**Figure 3:**
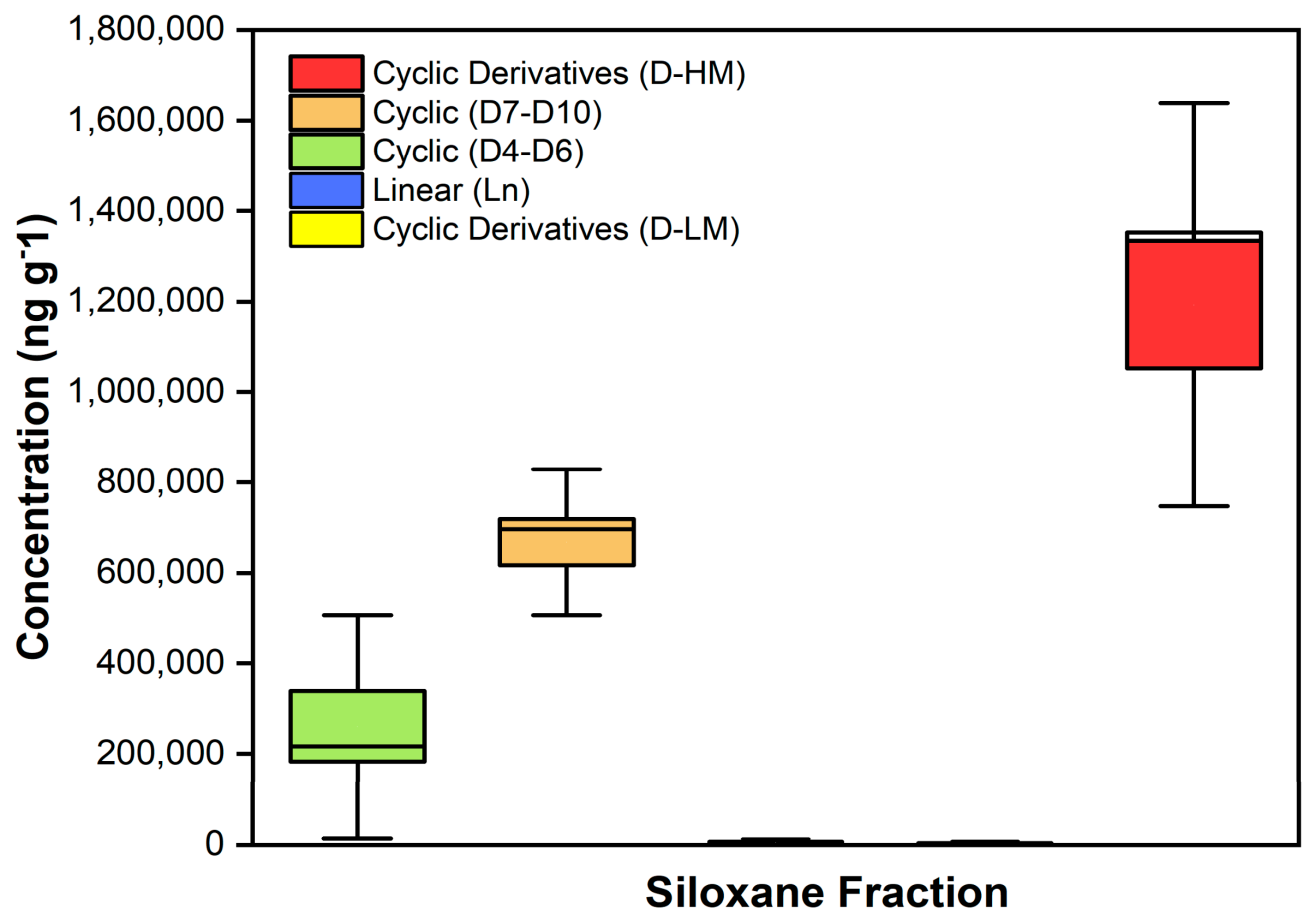
Distribution of siloxane fractions in simulant E (Tenax). Concentrations are expressed as the sum of all compounds assigned to each fraction.

To this end, siloxanes were classified into five fractions: low-molecular-weight cyclic siloxanes (D4–D6), high-molecular-weight cyclic siloxanes (D7–D10), low- and high-molecular-weight Dn-type derivatives (D-LM and D-HM), and linear siloxanes (Ln) (Figure 3). For each sample, the total concentration of every siloxane fraction was obtained by summing the concentrations of all individual compounds assigned to that group. The resulting values were then used to generate the box-and-whisker plots.

Migration was dominated by the D-HM and D7–D10 fractions. High-molecular-weight cyclic derivatives (D-HM) exhibited the highest median, around 1.3 × 10^6^ ng g^−1^, followed by D7–D10 cyclic siloxanes, close to 7.0 × 10^5^ ng g^−1^. In contrast, low-molecular-weight cyclic siloxanes (D4–D6) were found at lower levels, on the order of 2.0–3.0 × 10^5^ ng g^−1^ and showed greater variability among samples. These results indicate that migration was not restricted to low-molecular-weight volatile species, but also involved larger cyclic oligomers retained within the elastomeric network, whose release may be promoted under the high-temperature conditions applied during the assay. The Ln and D-LM fractions showed the lowest concentrations and limited variability among samples, consistent with a lower proportion of these compounds in the silicone matrix.

Notably, D6 individually exceeded the generic specific migration limit of 60 mg kg^−1^ established in Spanish Royal Decree 847/2011 for substances without an established SML or other restriction in six of the ten samples (Table S5), reaching a maximum concentration of 546.6 mg kg^−1^ in EM18. In addition, the sum of D4–D10 exceeded this value in all samples. These results confirm the substantial contribution of cyclic siloxanes to the migration profile of the studied silicone molds.

## 4. Conclusions

The combination of headspace analysis and migration tests enabled a comprehensive characterization of the released compounds. The resulting profile revealed a wide variety of substances, including siloxanes and NIASs, some of which were released with repeated use. The highest concentrations were observed in the solid simulant, whereas quantification in aqueous media was limited. In the latter, the chemical profile varied between the first and third migrations, with a decrease in the number of initially detected substances and the appearance of additional substances in the third migration. Concentrations determined in the third migration remained below the regulatory and toxicological criteria considered.

The silicone molds evaluated showed a high release of VOCs and cyclic siloxane oligomers. In all samples, the sum of cyclic siloxanes determined in simulant E exceeded the generic specific migration limit of 60 mg kg^−1,^ and the VOC loss exceeded the 0.5% threshold recommended by the BfR.

Variability among the samples, along with the detection of NIASs such as phthalates, suggests differences in the composition and quality of commercial products, without a clear correlation with labeling information. The results highlight the need for greater control over manufacturing processes and more specific criteria for evaluating the migration and quality of these materials.

## Figures and Tables

**Table 1:** Characteristics of the analyzed silicone molds.

Code	Color	Maximum Use Temperature	Origin	Food-Contact Information
DM15	Green	Not specified	China	Fork-and-cup symbol
DM25	Black	230 °C	China	Fork-and-cup symbol
EM18	Bluish gray	Not specified	Not specified	Not specified
EM20	Red	Not specified	Not specified	Not specified
JM10	Gray	Not specified	Not specified	Not specified
JM20	Lilac	Not specified	Not specified	Not specified
JM24	Purple	230 °C	China	Fork-and-cup symbol
JM25	Red	Not specified	Not specified	Not specified
TM17	Pink	High-temperature resistant	Not specified	Food grade
TM18	Gray	230 °C	China	Fork-and-cup symbol

Not specified: information not provided on the product label. The fork-and-cup symbol indicates suitability for food contact in accordance with Regulation (EC) No. 1935/2004 [21].

**Table 2:** Non-targeted identification of volatile compounds detected in migration assays and headspace analysis of silicone molds.

No.	Compound	Molecular Formula	CAS	RT	HS1	HS3	Simulant					RI_e_	RI_l_	DF	CC	SML (mg kg^−1^)
							A1	A3	B1	B3	E					
1	ethylbenzene *	C_8_H_10_	100-41-4	6.07	✓	✓						861	864	8	I	--
2	2-Heptanone	C_7_H_14_O	110-43-0	6.87		✓						889	893	1	II	--
3	styrene *	C_8_H_8_	100-42-5	6.91	✓	✓						890	894	10	I	No SML
4	heptanal	C_7_H_14_O	111-71-7	7.17		✓						899	899	1	I	--
5	propylbenzene *	C_9_H_12_	103-65-1	8.50	✓	✓						956	950	6	I	--
6	benzaldehyde *	C_7_H_6_O	100-52-7	8.68	✓							963	961	1	I	No SML
7	cyclosiloxane derivative (class D3)			9.18	✓	✓	✓					984	--	7	--	--
8	2-Octanone	C_8_H_16_O	111-13-7	9.34		✓						991	992	2	II	--
9	tetraethyl orthosilicate	C_8_H_20_O_4_Si	78-10-4	9.40							✓	997	--	5	--	--
10	octamethylcyclotetrasiloxane (D4) *	C_8_H_24_O_4_Si_4_		9.56	✓	✓	✓	✓			✓	1005	994	10	--	--
11	octanal	C_8_H_16_O	124-13-0	9.64							✓	1009	1002	1	I	--
12	2-ethylhexan-1-ol	C_8_H_18_O	104-76-7	10.17	✓	✓	✓	✓	✓	✓		1031	1025	10	I	30
13	eucalyptol	C_10_H_18_O	470-82-6	10.19	✓	✓						1033	1031	3	III	--
14	trans-β-Ocimene	C_10_H_16_	3779-61-1	10.32		✓						1039	1048	1	I	--
15	(Z)-3,7-dimethyl-1,3,6-octatriene	C_10_H_16_	3338-55-4	10.52		✓						1050	1041	1	I	--
16	3-carene	C_10_H_16_	13466-78-9	10.54	✓							1050	1011	1	I	--
17	4-methyldecane	C_11_H_24_	2847-72-5	10.75	✓	✓						1061	1061	1	I	--
18	acetophenone	C_8_H_8_O	98-86-2	10.84	✓	✓	✓		✓	✓	✓	1066	1065	10	I	--
19	cyclosiloxane derivative (class D3)			11.11	✓	✓	✓	✓			✓	1080		10	--	--
20	2-phenylpropan-2-ol	C_9_H_12_O	617-94-7	11.24	✓	✓						1086	1080	1	I	--
21	bis(trimethylsilyl) diethyl orthosilicate	C_10_H_28_O_4_Si_3_	3555-45-1	11.28			✓				✓	1094		8	III	--
22	2-Nonanone	C_9_H_18_O	821-55-6	11.34		✓						1091	1093	1	II	--
23	undecane *	C_11_H_24_	1120-21-4	11.48	✓	✓						1098	1100	1	I	--
24	nonanal *	C_9_H_18_O	124-19-6	11.60	✓	✓	✓		✓	✓		1105	1108	10	I	--
25	1,1,3,3,5,5,7,7,9,9-decamethylpentasiloxane (L5)	C_10_H_32_O_4_Si_5_	995-83-5	11.79							✓	1124		1	--	--
26	4-ethyldecane	C_12_H_26_	1636-44-8	12.06	✓	✓						1140	1152	1	I	--
27	decamethylcyclopentasiloxane (D5) *	C_10_H_30_O_5_Si_5_	541-02-6	12.44	✓	✓	✓	✓			✓	1163		10	--	--
28	2-methylundecane	C_12_H_26_	7045-71-8	12.57	✓	✓						1171	1165	3	I	--
29	3-methylundecane	C_12_H_26_	1002-43-3	12.68	✓	✓						1178	1171	4	I	--
30	naphthalene *	C_10_H_8_	91-20-3	13.00			✓		✓	✓		1189	1182	7	III	--
31	2-Decanone	C_10_H_20_O	693-54-9	13.05		✓						1192	1193	1	II	--
32	α-terpineol (isomer)	C_10_H_18_O	98-55-5	13.10			✓	✓	✓			1194	1198	3	III	--
33	dodecane *	C_12_H_26_	112-40-3	13.17	✓	✓						1199	1200	10	I	--
34	decanal	C_10_H_20_O	112-31-2	13.28			✓				✓	1206	1203	3	I	--
35	cyclosiloxane derivative (class D4)			13.63	✓	✓	✓	✓			✓	1230		10	--	--
36	cyclosiloxane derivative (class D4)			14.11			✓	✓				1262		10	--	--
37	nonanoic acid	C_9_H_18_O_2_	112-05-0	14.24					✓	✓		1271	1280	8	I	--
38	isobornyl acetate (isomers)	C_12_H_20_O_2_	125-12-2	14.58	✓	✓	✓	✓	✓			1293	1294	4	I	--
39	tridecane *	C_13_H_28_	629-50-5	14.65	✓	✓						1299	1300	2	I	--
40	undecanal *	C_11_H_22_O	112-44-7	14.66							✓	1308	1304	1	I	--
41	1-methylnaphthalene	C_11_H_10_	90-12-0	14.70	✓							1302	1313	3	III	--
42	cyclosiloxane derivative (class D4)			14.81	✓							1310		6	--	--
43	dodecamethylcyclohexasiloxane (D6) *	C_12_H_36_O_6_Si_6_	540-97-6	15.03	✓	✓	✓	✓			✓	1326		10	--	--
44	2-methylpropyl 3-hydroxy-2,2,4-trimethylpentanoate	C_12_H_24_O_3_	244074-78-0	15.48	✓		✓	✓	✓	✓		1359		7	II	--
45	4-tert-butylcyclohexyl acetate	C_12_H_22_O_2_	32210-23-4	15.71			✓	✓	✓			1375	1368	1	II	--
46	tetradecane *	C_14_H_30_	629-59-4	16.01	✓							1397	1400	1	I	--
47	diphenyl ether *	C_12_H_10_O	101-84-8	16.18			✓		✓			1410	1396	3	III	--
48	2,4,7,9-tetramethyl-5-decyn-4,7-diol	C_14_H_26_O_2_	126-86-3	16.27			✓		✓			1416	1407	2	III	--
49	cyclosiloxane derivative (class D5)			16.47			✓					1433		9	--	--
50	diisopropyl adipate	C_12_H_22_O_4_	6938-94-9	16.72				✓	✓			1452	1464	1	I	--
51	2,4-dimethylquinoline	C_11_H_11_N	1198-37-4	16.75			✓					1454	1454	2	III	--
52	2-methoxynaphthalene	C_11_H_10_O	93-04-9	16.84					✓			1461	1447	1	III	--
53	dimethyl phthalate *	C_10_H_10_O_4_	131-11-3	16.84			✓				✓	1461	1461	8	I	--
54	undecanoic acid	C_11_H_22_O_2_	112-37-8	16.87					✓	✓		1464	1465	1	I	--
55	5-hexyldihydro-2(3h)-furanone	C_10_H_18_O_2_	706-14-9	16.99					✓		✓	1474	1470	1	II	--
56	2,6-Di-tert-butyl-4-hydroxy-4-methylcyclohexa-2,5-dien-1-one	C_15_H_24_O_2_	10396-80-2	17.02	✓		✓	✓	✓	✓		1476	1478	5	II	--
57	1-[4-(1-hydroxy-1-methylethyl)phenyl]ethanone	C_11_H_14_O_2_	54549-72-3	17.12			✓					1481	1432	1	I	--
58	4-(2,6,6-trimethyl-1-cyclohexen-1-yl)-3-buten-2-one	C_13_H_20_O	14901-07-6	17.25			✓	✓	✓	✓		1494	1488	6	I	--
59	tetradecamethylcycloheptasiloxane (D7)	C_14_H_42_O_7_Si_7_	107-50-6	17.30	✓	✓	✓	✓			✓	1498		10	--	--
60	tridecanal	C_13_H_26_O	10486-19-8	17.33							✓	1512	1512	2	I	--
61	1,2,3,5,6,7-hexahydro-1,1,2,3,3-pentamethyl-4h-inden-4-one	C_14_H_22_O	33704-61-9	17.49			✓	✓				1514	1503	4	III	--
62	2,4-di-tert-butylphenol *	C_14_H_22_O	96-76-4	17.51			✓	✓				1515	1513	2	I	--
63	butylated hydroxytoluene (BHT)	C_15_H_24_O	128-37-0	17.57			✓					1521	1514	2	II	3
64	dibutyl but-2-enedioate	C_12_H_20_O_4_	105-76-0	17.80			✓	✓	✓	✓		1539		5	I	--
65	1,1,3,3,5,5,7,7,9,9,11,11,13,13-tetradecamethylheptasiloxane (L7)	C_14_H_44_O_6_Si_7_	19095-23-9	17.91			✓	✓				1548		10	--	--
66	dodecanoic acid	C_12_H_24_O_2_	143-07-7	18.06			✓		✓	✓		1561	1571	7	I	No SML
67	5-heptyldihydro-2(3H)-Furanone	C_11_H_20_O_2_	104-67-6	18.29			✓		✓	✓		1580	1573	7	II	--
68	hexadecane	C_16_H_34_	544-76-3	18.38							✓	1600	1600	1	I	--
69	diethyl phthalate *	C_12_H_14_O_4_	84-66-2	18.54			✓	✓	✓	✓		1600	1585	10	I	--
70	tetradecanal	C_14_H_28_O	124-25-4	18.55							✓	1615	1611	1	I	--
71	2,2,4-trimethyl-1,3-pentanediol diisobutyrate *	C_16_H_30_O_4_	6846-50-0	18.56		✓	✓	✓				1603	1588	2	I	5
72	cedrol	C_15_H_26_O	77-53-2	18.79			✓	✓	✓	✓		1622	1604	8	III	--
73	benzophenone *	C_13_H_10_O	119-61-9	19.02			✓	✓	✓	✓		1643	1625	10	III	0.6
74	methyl 3-oxo-2-pentylcyclopentaneacetate	C_13_H_22_O_3_	24851-98-7	19.22			✓	✓	✓	✓	✓	1661	1648	9	II	--
75	hexadecamethylcyclooctasiloxane (D8)	C_16_H_48_O_8_Si_8_	556-68-3	19.30	✓	✓	✓	✓			✓	1668		10	--	--
76	γ-dodecalactone	C_12_H_22_O_2_	2305-05-7	19.52			✓		✓	✓	✓	1687	1673	1	II	--
77	2-benzylideneoctanal	C_15_H_20_O	101-86-0	20.29				✓	✓			1759	1750	1	I	--
78	tetradecanoic acid	C_14_H_28_O_2_	544-63-8	20.38			✓	✓	✓	✓		1767	1780	9	I	No SML
79	(4-methylphenyl)phenylmethanone	C_14_H_12_O	134-84-9	20.45			✓		✓			1774		2	III	--
80	benzyl benzoate	C_14_H_12_O_2_	120-51-4	20.52			✓	✓	✓			1781	1765	8	I	--
81	octadecane	C_18_H_38_	593-45-3	20.59							✓	1799	1800	1	I	--
82	9-methylidenefluorene	C_14_H_10_	4425-82-5	20.76			✓					1803		1	III	--
83	hexadecanal	C_16_H_32_O	629-80-1	20.80							✓	1818	1817	3	I	--
84	2-phenoxyethoxybenzene	C_14_H_14_O_2_	104-66-5	20.90			✓	✓	✓	✓		1817	1811	10	III	--
85	(4-chlorophenyl)phenylmethanone	C_13_H_9_ClO	134-85-0	20.99			✓		✓	✓		1825		2	III	--
86	octadecamethylcyclononasiloxane (D9)	C_18_H_54_O_9_Si_9_	556-71-8	21.04	✓	✓	✓				✓	1830		10	--	--
87	pentadecanoic acid	C_15_H_30_O_2_	1002-84-2	21.37			✓		✓	✓		1862	1867	7	I	--
88	1-hexadecanol	C_16_H_34_O	36653-82-4	21.46							✓	1883	1882	4	--	No SML
89	1-(3-ethyl-5,5,8,8-tetramethyl-6,7-dihydronaphthalen-2-yl)ethanone	C_18_H_26_O	88-29-9	21.47	✓	✓	✓	✓	✓	✓		1872		6	I	--
90	diisobutyl phthalate *	C_16_H_22_O_4_	84-69-5	21.53			✓	✓	✓	✓		1878	1873	8	I	--
91	1,1,1,3,3,5,5,7,7,9,9,11,11,13,13,15,15,17,17,17-icosamethylnonasiloxane (L9)	C_20_H_60_O_8_Si_9_	2652-13-3	21.56							✓	1893		1	I	--
92	2-heptadecanone	C_17_H_34_O	2922-51-2	21.67							✓	1904	1901	1	II	--
93	heptadecanal	C_17_H_34_O	629-90-3	21.84							✓	1921	1937	1	I	--
94	7,9-di-tert-butyl-1-oxaspiro(4,5)deca-6,9-diene-2,8-dione	C_17_H_24_O_3_	82304-66-3	22.09			✓	✓	✓	✓		1933	1929	10	III	--
95	diphenyl sulfone	C_12_H_10_O_2_S	127-63-9	22.21					✓			1946		2	III	3
96	n-hexadecanoic acid	C_16_H_32_O_2_	57-10-3	22.35			✓	✓	✓	✓	✓	1960	1972	10	I	No SML
97	eicosamethylcyclodecasiloxane (D10)	C_20_H_60_O_10_Si_10_	18772-36-6	22.45							✓	1983		10	I	--
98	dibutyl phthalate *	C_16_H_22_O_4_	84-74-2	22.47				✓	✓	✓	✓	1972	1970	8	I	0.3
99	1,1,3,3,5,5,7,7,9,9,11,11,13,13,15,15-hexadecamethyloctasiloxane (L8)	C_16_H_50_O_7_Si_8_	19095-24-0	22.56	✓	✓						1982		10	--	--
100	3,5-di-tert-Butyl-4-hydroxyphenylpropionic acid *	C_17_H_26_O_3_	20170-32-5	22.77					✓			2003		1	II	--
101	octadecanal	C_18_H_36_O	638-66-4	22.84							✓	2024	2021	2	I	--
102	isopropyl palmitate	C_19_H_38_O_2_	142-91-6	22.99						✓		2026	2011	4	I	--
103	1,1,1,3,3,5,5,7,7,9,9,11,11,13,13,15,15,17,17,19,19,19-docosamethyldecasiloxane (L10)	C_22_H_66_O_9_Si_10_	556-70-7	23.07							✓	2048		3	--	--
104	1-octadecanol	C_18_H_38_O	112-92-5	23.40			✓				✓	2070	2082	3	I	--
105	2-[(Z)-octadec-9-enoxy]ethanol	C_20_H_40_O_2_	5353-25-3	23.66			✓			✓	✓	2097		1	I	--
106	5-dodecyldihydro- 2(3H)-furanone	C_16_H_30_O_2_	730-46-1	23.80			✓			✓	✓	2113	2106	3	II	--
107	cyclosiloxane derivative (class D10)			23.88							✓	2121		9	--	--
108	oleic acid	C_18_H_34_O_2_	112-80-1	24.09			✓					2145	2152	10	I	No SML
109	octadecanoic acid *	C_18_H_36_O_2_	57-11-4	24.29			✓		✓	✓	✓	2167	2172	10	I	No SML
110	butyl hexadecanoate	C_20_H_40_O_2_	111-06-8	24.34							✓	2187	2188	3	I	No SML
111	ethyl octadecanoate	C_20_H_40_O_2_	111-61-5	24.41							✓	2194	2194	2	I	--
112	linear siloxane derivative (L10)			24.44							✓	2199		2	--	--
113	hexadecanamide	C_16_H_33_NO	629-54-9	24.49			✓				✓	2190	2182	1	III	--
114	cyclosiloxane derivative (class D10)			24.80							✓	2240		2	I	--
115	cyclosiloxane derivative (class D10)			25.16							✓	2282		10	--	--
116	methyl 4-hydroxyoctadecanoate	C_19_H_38_O_3_	2420-38-4	25.52							✓	2324		3	II	--
117	linear siloxane derivative (L10)			25.72							✓	2348		4	--	--
118	(Z)-octadec-9-enamide	C_18_H_35_NO	301-02-0	25.89			✓				✓	2369	2375	4	III	No SML
119	butyl octadecanoate	C_22_H_44_O_2_	123-95-5	26.05							✓	2387	2388	3	I	No SML
120	cyclosiloxane derivative (class D10)			26.36							✓	2426		10		--
121	cyclosiloxane derivative (class D10)			27.15							✓	2527		3		--
122	linear siloxane derivative (L10)			28.01							✓	2640		3		--
123	cyclosiloxane derivative (class D10)			28.54							✓	2712		9		--
124	cyclosiloxane derivative (class D10)			29.51							✓	2849		10		--
125	cyclosiloxane derivative (class D10)			30.45							✓	2983		10		--
126	cyclosiloxane derivative (class D10)			31.49							✓	3117		10		--
127	cyclosiloxane derivative (class D10)			32.76							✓			10		--
128	cyclosiloxane derivative (class D10)			34.33							✓			10		--

✓: compound detected; *: compound confirmed with an analytical standard; HS: headspace; A: simulant A; B: simulant B; E: solid simulant; numbers 1 and 3 indicate migration or heating cycles; RT: retention time; RIe: experimental retention index; RIl: retention index reported in the literature; DF: detection frequency (number of samples in which the compound was detected relative to the total analyzed, n = 10); CC: Cramer class assigned using Toxtree; specific migration limit (mg kg^−1^); --: not available (RIl) or not assigned (Cramer Changes occurring across successive migration cycles may be associated with different processes. On the one hand, the release of readily accessible siloxanes and other mobile compounds during the initial contacts progressively reduces the fraction available for subsequent migration. At the same time, environmental VOCs previously absorbed by the silicone may subsequently be released from the material, while thermal exposure may promote the transformation or degradation of formulation-related substances [27,29,32,33]. The evolution of the chemical profile during repeated use may result from a combination of several of these processes. The appearance of substances in subsequent migration cycles may result either from their formation during thermal exposure or from the delayed release of compounds retained within the silicone matrix. Consequently, the changes observed across migration cycles may simultaneously involve depletion, release, and formation processes.

## Data Availability

The data supporting the results reported in this study are available in the Supplementary Materials. Additional data are available from the corresponding author upon reasonable request.

## Supplementary Materials

The following supporting information can be downloaded at: https://www.mdpi.com/article/10.3390/polym18172162/s1, Figure S1: Silicone molds for use in air fryers; Figure S2: Silicone specimens used in the migration tests after cutting; Table S1: Analytical parameters of the SPME-GC-MS method: working range, linearity, LOD, LOQ, and codes assigned to the available standards used for quantification; Table S2: Analytical parameters of the GC-MS method: working range, linearity, LOD, LOQ, and codes assigned to the available standards used for quantification; Table S3: Migration results of selected compounds in simulant A, expressed in ng g^−1^ (first and third migration, Part I and II); Table S4: Migration results of selected compounds in simulant B, expressed in ng g^−1^ (first and third migration, Part I and II); Table S5: Migration results of selected compounds in simulant E (Tenax), expressed in mg kg^−1^.
