# Supplementary material for: Chemical Characterization and Migration Profile of Silicone Molds for Air Fryers
Source: Polymers (Basel). 2026 Sep 4;18(17):2162. doi: 10.3390/polym18172162 (PMC13568349; doi:10.3390/polym18172162)
Supplement: Supplementary file 1 [file polymers-18-02162-s001.zip › polymers-4469706-supplementary.pdf]

Chemical Characterization and Migration Profile of Silicone Molds for Air Fryers

Janira Jaén [1,2,*,†], Cristhopher Castillo [1,†], Lourdes Arjona [3], Paula Vera [4], Elena Canellas [4], Cristina Nerín [4], Jorge González-Chan [1], Felipe Barria-Almengor [1], Gerardo Cáceres [3] and Carlos H. Ríos Martínez [3,†]

[1] Analytical Chemistry Department, Universidad de Panamá, Simón Bolivar Avenue, P.O. Box 3366, Panama, Panama; cristhopher.castillo@up.ac.pa (C.C.); jorge.gonzalezc@up.ac.pa (J.G.-C.); feli-pe.barria@up.ac.pa (F.B.-A.)
[2] Sistema Nacional de Investigadores (SNI), SENACYT, Panama, Panama
[3] Organic Chemistry Department, Universidad de Panamá, Simón Bolivar Avenue,P.O. Box 3366, Panama, Panama; lourdes.arjona@up.ac.pa (L.A.); gerardo.caceres@up.ac.pa (G.C.); carlos.rios-m@up.ac.pa (C.H.R.M.)
[4] Analytical Chemistry Department, GUIA Group, I3A, EINA, University of Zaragoza, Mª de Luna 3, 50018 Zaragoza, Spain; pvera@unizar.es (P.V.); elenac@unizar.es (E.C.); cnerin@unizar.es (C.N.)
[*] Correspondence: janira.jaen@up.ac.pa; Tel.: +507-5236227
[†] These authors contributed equally to this work.

Figures

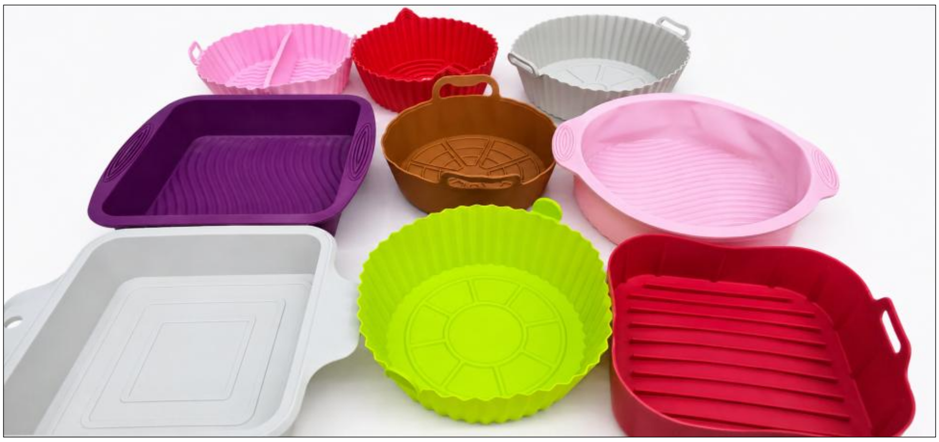

Figure S1. Silicone molds for use in air fryers.

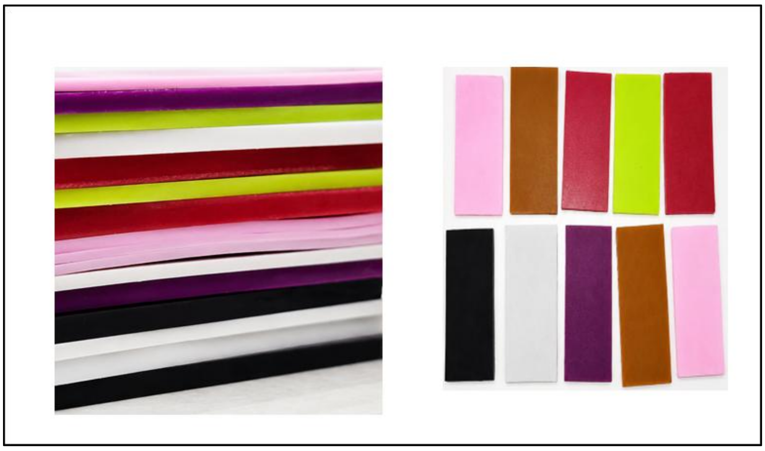

Figure S2. Silicone specimens used in the migration tests after cutting.

Tables

Table S1. Analytical parameters of the SPME-GC-MS method: working range, linearity, LOD, LOQ, and codes assigned to the available standards used for quantification

Code	Compound	Working range (ng g^{-1})	R2	LOD (ng g^{-1})	LOQ (ng g^{-1})
a	Octamethylcyclotetrasiloxane D4	1-588	0.9925	0.19	0.62
b	decamethyltetrasiloxane L4	1-486	0.9927	0.28	0.94
c	decamethylcyclopentasiloxane D5	1-574	0.9907	0.11	0.35
d	dodecamethylcyclohexasiloxane	1-527	0.9926	0.13	0.42
e	dimethyl phthalate (DMP)	1-502	0.9974	0.33	1.10
f	dibutyl phthalate	1-505	0.9925	0.31	1.03
g	diisobutyl phthalate	1-507	0.9904	0.35	1.17
h	benzophenone	1-535	0.9916	0.23	0.75
i	naphthalene	1-509	0.9936	0.23	0.78
j	diphenyl sulfone	1-501	0.9947	0.29	0.97
k	diphenyl ether	1-513	0.9898	0.24	0.81
l	2,4-di-tert-butylphenol	1-524	0.9888	0.14	0.45
m	2,2,4-trimethyl-1,3-pentanediol diisobutyrate	1-530	0.9901	0.12	0.40
n	2,6-di-tert-butyl-1,4-benzoquinone	1-515	0.9933	0.23	0.76
o	hexadecanoic acid	1-528	0.9939	0.32	1.08
p	dodecan-1-ol	1-516	0.9918	0.35	1.15
q	methyl oleate	1-482	0.9940	0.12	0.41

R^2: coefficient of determination. LOD: limit of detection. LOQ: limit of quantification.

Table S2. Analytical parameters of the GC-MS method: working range, linearity, LOD, LOQ, and codes assigned to the available standards used for quantification

Code	Compound	Working range (mg kg^{-1})	R2	LOD (mg kg^{-1})	LOQ (mg kg^{-1})
a	Octamethylcyclotetrasiloxane D4	0.05-7.2	0.9993	0.00020	0.00068
b	decamethyltetrasiloxane L4	0.05-6.8	0.9999	0.0014	0.0048
c	decamethylcyclopentasiloxane D5	0.01-15.3	0.9980	0.0067	0.022
d	dodecamethylcyclohexasiloxane	0.03-22.9	0.9985	0.0013	0.0045
e	dimethyl phthalate (DMP)	0.05-7.4	0.9978	0.0028	0.0092
f	dibutyl phthalate	0.05-7.3	0.9941	0.0043	0.015

R^2: coefficient of determination. LOD: limit of detection. LOQ: limit of quantification

Table S3. Migration results of selected compounds in simulant A, expressed in ng g⁻¹ (first and third migration, Part I)

No	Compound	Migration concentration (ng g⁻¹)										QS
		DM15		DM25		EM18		EM20		JM10		
		A1	A3	A1	A3	A1	A3	A1	A3	A1	A3	
1	cyclosiloxane derivative (class D3)	ND	ND	ND	ND	ND	ND	ND	ND	ND	ND	a
2	octamethylcyclotetrasiloxane (D4) *	<LOQ	<LOQ	<LOQ	<LOQ	<LOQ	<LOQ	<LOQ	<LOQ	<LOQ	<LOQ	a
3	2-ethylhexan-1-ol	ND	ND	ND	<LOQ	<LOQ	<LOQ	ND	ND	ND	ND	p
4	cyclosiloxane derivative ((class D3, RT= 11.11)	<LOQ	<LOQ	<LOQ	<LOQ	<LOQ	<LOQ	<LOQ	<LOQ	<LOQ	<LOQ	a
5	bis(trimethylsilyl) diethyl orthosilicate	ND	ND	ND	ND	<LOQ	ND	<LOQ	ND	ND	ND	b
6	decamethylcyclopentasiloxane (D5)*	<LOQ	ND	<LOQ	<LOQ	<LOQ	<LOQ	<LOQ	<LOQ	<LOQ	<LOQ	c
7	naphthalene*	<LOQ	<LOD	<LOD	<LOD	<LOQ	<LOD	<LOQ	<LOD	<LOQ	<LOD	i
8	α-terpineol (isomer)	ND	ND	ND	ND	ND	ND	ND	ND	ND	ND	p
9	cyclosiloxane derivative (class D4, RT=13.63)	<LOQ	<LOQ	<LOQ	<LOQ	<LOQ	<LOQ	<LOQ	<LOQ	<LOQ	<LOQ	a
10	cyclosiloxane derivative ((class D4, RT=14.11)	<LOQ	<LOQ	<LOQ	<LOQ	<LOQ	<LOQ	<LOQ	<LOQ	<LOQ	<LOQ	a
11	dodecamethylcyclohexasiloxane (D6)*	<LOQ	<LOD	<LOQ	<LOQ	<LOQ	<LOQ	<LOQ	<LOQ	<LOQ	<LOQ	d
12	diphenyl ether*	<LOD	<LOD	<LOQ	<LOD	<LOQ	<LOD	<LOD	<LOD	<LOD	<LOD	k
13	2,4,7,9-tetramethyl-5-decyn-4,7-diol	ND	ND	ND	ND	ND	ND	ND	ND	ND	ND	m
14	cyclosiloxane derivative (class D5, RT=16.47)	<LOQ	ND	<LOQ	ND	<LOQ	ND	<LOQ	ND	<LOQ	ND	c
15	2,4-dimethylquinoline	ND	ND	ND	ND	ND	ND	ND	ND	<LOQ	ND	i
16	2-methoxynaphthalene	ND	ND	ND	ND	ND	ND	ND	ND	ND	ND	i
17	dimethyl phthalate*	6.3±1.5	<LOD	49.1±6.7	<LOD	2.3±0.2	<LOQ	19.9±1.4	<LOD	16.2±2.2	<LOD	e
18	tetradecamethylcycloheptasioxane (D7)	<LOQ	<LOQ	<LOQ	<LOQ	<LOQ	<LOQ	<LOQ	<LOQ	<LOQ	<LOQ	c

No	Compound	Migration concentration (ng g^{-1})										QS
		DM15		DM25		EM18		EM20		JM10		
		A1	A3	A1	A3	A1	A3	A1	A3	A1	A3	
19	1,2,3,5,6,7-hexahydro-1,1,2,3,3-pentamethyl-4h-inden-4-one	ND	ND	<LOQ	<LOQ	ND	ND	<LOQ	<LOQ	ND	ND	h
20	butylated hydroxytoluene (BHT)	ND	ND	<LOQ	ND	ND	ND	ND	ND	ND	ND	l
21	1,1,3,3,5,5,7,7,9,9,11,11,13,13-tetradecamethylheptasiloxane (L7)	<LOQ	<LOQ	<LOQ	<LOQ	<LOQ	<LOQ	<LOQ	<LOQ	<LOQ	<LOQ	b
22	diethyl phthalate*	51.4±10.5	1.4±0.1	90.3±14.2	3.8±0.2	5.2±2.9	1.2±0.4	186.6±7.2	1.2±0.3	<LOQ	<LOQ	e
23	2,2,4-trimethyl-1,3-pentanediol diisobutyrate *	<LOD	<LOD	<LOD	<LOD	<LOD	<LOD	<LOQ	<LOQ	<LOD	<LOD	m
24	benzophenone*	<LOQ	<LOQ	<LOQ	<LOQ	<LOQ	<LOQ	1.9±0.8	<LOQ	<LOQ	<LOQ	h
25	hexadecamethylcyclooctasiloxane (D8)	<LOQ	<LOQ	<LOQ	<LOQ	<LOQ	<LOQ	<LOQ	<LOQ	<LOQ	<LOQ	c
26	(4-methylphenyl)phenylmethanone	ND	ND	<LOQ	ND	ND	ND	<LOQ	ND	ND	ND	h
27	9-methylidenefluorene	ND	ND	ND	ND	ND	ND	ND	ND	<LOQ	ND	i
28	2-phenoxyethoxybenzene	<LOQ	<LOQ	<LOQ	<LOQ	<LOQ	<LOQ	<LOQ	<LOQ	<LOQ	<LOQ	k
29	(4-chlorophenyl)phenylmethanone	<LOQ	ND	<LOQ	ND	ND	ND	<LOQ	ND	ND	ND	h
30	octadecamethylcyclononasiloxane (D9)	<LOQ	ND	ND	ND	ND	ND	ND	ND	<LOQ	ND	c
31	diisobutyl phthalate*	<LOQ	<LOQ	<LOQ	<LOQ	<LOD	<LOD	<LOQ	<LOQ	<LOD	<LOD	g
32	7,9-di-tert-butyl-1-oxaspiro(4,5)deca-6,9-diene-2,8-dione	ND	ND	ND	ND	ND	ND	<LOQ	ND	<LOQ	ND	n
33	dibutyl phthalate*	46.7±13.7	<LOQ	<LOD	<LOQ	29.4±5.2	<LOQ	13.5±1.3	<LOQ	<LOD	<LOD	f
34	hexadecanamide	43.0±8.5	ND	ND	ND	ND	ND	<LOQ	ND	5.7±1.0	ND	o
35	(Z)-octadec-9-enamide	53.1±2.8	ND	ND	ND	ND	ND	<LOQ	ND	<LOQ	ND	q

A1 and A3: first and third migration, respectively; <LOD: below the limit of detection; <LOQ: below the limit of quantification; ND = Not detected; QS: quantification standard used (see Table S1); *: compound with an available analytical standard.

Table S3. Migration results of selected compounds in simulant A, expressed in ng g^{-1} (first and third migration, Part II)

No	Compound	Migration concentration (ng g^{-1})										QS
		JM20		JM24		JM25		TM17		TM18		
		A1	A3	A1	A3	A1	A3	A1	A3	A1	A3	
1	cyclosiloxane derivative (class D3)	ND	ND	ND	ND	ND	ND	<LOQ	ND	ND	ND	a
2	octamethylcyclotetrasiloxane (D4) *	<LOQ	<LOQ	<LOQ	<LOQ	<LOQ	<LOQ	<LOQ	<LOQ	<LOQ	<LOQ	a
3	2-ethylhexan-1-ol	ND	ND	ND	ND	ND	<LOQ	<LOQ	ND	ND	<LOQ	p
4	cyclosiloxane derivative (class D3)	<LOQ	<LOQ	<LOQ	<LOQ	ND	ND	ND	ND	<LOQ	<LOQ	a
5	bis(trimethylsilyl) diethyl orthosilicate	ND	ND	ND	ND	<LOQ	ND	<LOQ	ND	ND	ND	b
6	decamethylcyclopentasiloxane (D5)*	<LOQ	<LOQ	<LOQ	<LOQ	<LOQ	<LOQ	<LOQ	<LOQ	<LOQ	<LOQ	c
7	naphthalene*	<LOQ	ND	<LOQ	ND	ND	ND	ND	ND	ND	ND	i
8	α-terpineol (isomer)	ND	ND	ND	ND	ND	ND	ND	ND	<LOQ	<LOQ	p
9	cyclosiloxane derivative (class D4)	<LOQ	<LOQ	<LOQ	<LOQ	<LOQ	<LOQ	<LOQ	<LOQ	<LOQ	<LOQ	a
10	cyclosiloxane derivative (class D4)	<LOQ	<LOQ	<LOQ	<LOQ	<LOQ	<LOQ	<LOQ	<LOQ	<LOQ	<LOQ	a
11	dodecamethylcyclohexasiloxane (D6)*	<LOD	<LOQ	<LOD	<LOQ	<LOQ	<LOQ	<LOD	<LOQ	<LOD	<LOQ	d
12	diphenyl ether*	<LOD	<LOD	<LOQ	<LOD	<LOD	<LOD	<LOD	<LOD	<LOD	<LOD	k
13	2,4,7,9-tetramethyl-5-decyn-4,7-diol	ND	ND	<LOQ	ND	ND	ND	ND	ND	ND	ND	m
14	cyclosiloxane derivative (class D5, RT=16.47)	<LOQ	ND	ND	ND	<LOQ	ND	<LOQ	ND	<LOQ	ND	c
15	2,4-dimethylquinoline	<LOQ	ND	ND	ND	ND	ND	ND	ND	ND	ND	i
16	2-methoxynaphthalene	ND	ND	<LOQ	ND	ND	ND	<LOQ	ND	ND	ND	i
17	dimethyl phthalate*	24.5 ±3.9	<LOD	8.1±2.0	<LOD	5.5±0.9	<LOD	<LOD	<LOD	90.6± 10.1	<LOD	e
18	tetradecamethylcycloheptasiloxane (D7)	<LOQ	<LOQ	<LOQ	<LOQ	<LOQ	<LOQ	<LOQ	<LOQ	<LOQ	<LOQ	c

No	Compound	Migration concentration (ng g^{-1})										QS
		JM20		JM24		JM25		TM17		TM18		
		A1	A3	A1	A3	A1	A3	A1	A3	A1	A3	
19	1,2,3,5,6,7-hexahydro-1,1,2,3,3-pentamethyl-4h-inden-4-one	ND	ND	ND	ND	<LOQ	<LOQ	ND	ND	<LOQ	ND	h
20	butylated hydroxytoluene (BHT)	ND	ND	ND	ND	ND	ND	ND	ND	<LOQ	ND	l
21	1,1,3,3,5,5,7,7,9,9,11,11,13,13-tetradecamethylheptasiloxane (L7)	<LOQ	<LOQ	<LOQ	<LOQ	<LOQ	<LOQ	<LOQ	<LOQ	<LOQ	<LOQ	b
22	diethyl phthalate*	243.1±3.0	2.8±0.2	216.0±11.4	4.0±0.3	108.0±11.1	2.8± 0.7	<LOD	<LOD	185.0±16.7	8.1±1.8	e
23	2,2,4-trimethyl-1,3-pentanediol diisobutyrate *	<LOD	<LOD	<LOD	<LOD	<LOD	<LOD	<LOD	<LOD	<LOD	<LOD	m
24	benzophenone*	<LOD	<LOD	<LOD	<LOD	<LOD	<LOD	<LOD	<LOD	<LOD	<LOD	h
25	hexadecamethylcyclooctasiloxane (D8)	<LOQ	<LOQ	ND	<LOQ	<LOQ	<LOQ	<LOQ	<LOQ	ND	ND	c
26	(4-methylphenyl)phenylmethanone	ND	ND	ND	ND	ND	ND	ND	ND	ND	ND	h
27	9-methylidenefluorene	ND	ND	ND	ND	ND	ND	ND	ND	ND	ND	i
28	2-phenoxyethoxybenzene	<LOQ	<LOQ	<LOQ	<LOQ	<LOQ	<LOQ	<LOQ	<LOQ	<LOQ	<LOQ	k
29	(4-chlorophenyl)phenylmethanone	16.1±3.5	ND	ND	ND	ND	ND	ND	ND	ND	ND	h
30	octadecamethylcyclononasiloxane (D9)	ND	ND	ND	ND	<LOQ	ND	ND	ND	ND	ND	c
31	diisobutyl phthalate*	100.5±1.4	<LOQ	144.4± 4.5	<LOQ	<LOQ	<LOQ	<LOD	<LOD	6.5±1.8	<LOD	g
32	7,9-di-tert-butyl-1-oxaspiro(4,5)deca-6,9-diene-2,8-dione	<LOQ	<LOQ	ND	ND	<LOQ	ND	<LOQ	ND	ND	ND	n
33	dibutyl phthalate*	167.6±3.9	<LOD	223.7±2.2	<LOQ	4.6±1.1	<LOQ	<LOD	<LOD	30.5±4.2	<LOD	f
34	hexadecanamide	ND	ND	ND	ND	ND	ND	ND	ND	ND	ND	o
35	(Z)-octadec-9-enamide	<LOQ	ND	ND	ND	ND	ND	ND	ND	ND	ND	q

A1 and A3: first and third migration, respectively; <LOD: below the limit of detection; <LOQ: below the limit of quantification; ND = Not detected; QS: quantification standard used (see Table S1); *: compound with an available analytical standard.

Table S4. Migration results of selected compounds in simulant B, expressed in ng g^{-1} (first and third migration, Part I)

No	Compound	Migration concentration (ng g^{-1})										QS
		DM15		DM25		EM18		EM20		JM10		
		B1	B3	B1	B3	B1	B3	B1	B3	B1	B3	
1	naphthalene*	<LOQ	<LOD	<LOQ	<LOQ	<LOQ	<LOQ	<LOQ	<LOD	<LOQ	<LOD	i
2	α-terpineol (isomer)	<LOQ	ND	ND	ND	ND	ND	<LOQ	ND	ND	ND	p
3	diphenyl ether*	<LOD	<LOD	<LOD	<LOD	<LOD	<LOD	<LOD	<LOD	<LOD	<LOD	k
4	2,4,7,9-tetramethyl-5-decyn-4,7-diol	ND	ND	<LOQ	ND	ND	ND	ND	ND	ND	<LOQ	m
5	2-methoxynaphthalene	ND	ND	ND	ND	ND	ND	ND	ND	ND	ND	i
6	dimethyl phthalate	5.4±0.4	<LOQ	21.2±1.8	<LOQ	4.3±1.2	<LOQ	6.2±0.6	<LOQ	5.0±0.4	<LOQ	e
7	diethyl phthalate*	20.6±2.4	<LOQ	66.4±3.7	<LOQ	<LOQ	<LOQ	35.6± 2.2	<LOQ	<LOD	<LOD	e
8	benzophenone*	<LOQ	<LOD	<LOQ	<LOQ	<LOQ	<LOQ	<LOQ	<LOQ	<LOQ	<LOD	h
9	(4-methylphenyl)phenylmethanone	ND	ND	ND	ND	ND	<LOQ	ND	ND	ND	ND	h
10	2-phenoxyethoxybenzene	<LOQ	ND	<LOQ	<LOQ	<LOQ	<LOQ	<LOQ	ND	<LOQ	ND	k
11	(4-chlorophenyl)phenylmethanone	ND	ND	<LOQ	<LOQ	ND	ND	ND	ND	ND	ND	h
12	diisobutyl phthalate*	<LOQ	<LOQ	1.3±0.1	5.7±0.8	<LOQ	<LOD	<LOD	<LOD	<LOD	<LOD	g
13	7,9-di-tert-butyl-1-oxaspiro(4,5)deca-6,9-diene-2,8-dione	<LOQ	<LOQ	<LOQ	<LOQ	ND	<LOQ	ND	ND	ND	<LOQ	n
14	diphenyl sulfone*	<LOQ	<LOD	<LOQ	30.4±3.7	<LOD	<LOD	<LOD	<LOD	<LOD	<LOQ	j
15	dibutyl phthalate*	<LOQ	<LOQ	30.4±0.8	<LOQ	10.3±3.2	7.1±1.7	7.8±0.4	<LOQ	<LOQ	<LOQ	f

B1 and B3: first and third migration, respectively; <LOD: below the limit of detection; <LOQ: below the limit of quantification; ND = Not detected; QS: quantification standard used (see Table S1); *: compound with an available analytical standard.

Table S4. Migration results of selected compounds in simulant B, expressed in ng g^{-1} (first and third migration, Part II)

N°	Compound	Migration concentration (ng g^{-1})										QS
		JM20		JM24		JM25		TM17		TM18		
		B1	B3	B1	B3	B1	B3	B1	B3	B1	B3	
1	naphthalene*	<LOQ	<LOQ	<LOQ	<LOQ	<LOD	<LOD	<LOD	<LOD	<LOD	<LOD	i
2	α-terpineol (isomer)	ND	ND	ND	ND	ND	ND	ND	ND	ND	ND	p
3	diphenyl ether*	<LOD	<LOD	<LOQ	<LOD	<LOD	<LOD	<LOD	<LOD	<LOD	<LOD	k
4	2,4,7,9-tetramethyl-5-decyn-4,7-diol	ND	ND	<LOQ	ND	ND	ND	ND	ND	ND	<LOQ	m
5	2-methoxynaphthalene	ND	ND	<LOQ	ND	ND	ND	ND	ND	ND	ND	i
6	dimethyl phthalate	11.0± 1.3	<LOD	6.5± 1.3	<LOD	5.7± 0.9	<LOD	<LOD	<LOD	28.3± 0.6	<LOD	e
7	diethyl phthalate*	57.7±5.5	<LOQ	52.3± 3.7	<LOQ	<LOQ	<LOQ	<LOD	<LOD	40.1±4.2	<LOQ	e
8	benzophenone*	<LOQ	<LOQ	<LOQ	<LOQ	<LOQ	<LOQ	<LOQ	<LOQ	<LOQ	<LOQ	h
9	(4-methylphenyl)phenylmethanone	ND	ND	ND	ND	ND	ND	ND	ND	ND	ND	h
10	2-phenoxyethoxybenzene	<LOQ	<LOQ	<LOQ	ND	<LOQ	ND	<LOQ	<LOQ	<LOQ	<LOQ	k
11	(4-chlorophenyl)phenylmethanone	ND	ND	ND	ND	ND	ND	ND	ND	ND	ND	h
12	diisobutyl phthalate*	2.6±0.4	25.2±1.4	3.2±0.2	61.9±0.3	<LOQ	<LOD	<LOD	<LOD	<LOQ	<LOD	g
13	7,9-di-tert-butyl-1-oxaspiro(4,5)deca-6,9-diene-2,8-dione	<LOQ	<LOQ	<LOQ	<LOQ	ND	<LOQ	ND	<LOQ	ND	ND	n
14	diphenyl sulfone	ND	ND	ND	ND	ND	ND	ND	20.3±3.4	ND	ND	j
15	dibutyl phthalate*	52.5±5.7	59.9±1.6	66.4±3.8	128.8± 1.3	<LOD	<LOQ	<LOd	<LOQ	13.2±1.6	5.4±1.3	f

B1 and B3: first and third migration, respectively; <LOD: below the limit of detection; <LOQ: below the limit of quantification; ND = Not detected; QS: quantification standard used (see Table S1); *: compound with an available analytical standard.

Table S5. Migration results of selected compounds in simulant E (Tenax), expressed in mg kg^{-1}.

No.	Compound	Migration concentration (mg kg^{-1})										QS
		DM15	DM25	EM18	EM20	JM10	JM20	JM24	JM25	TM17	TM18	
1	tetraethyl orthosilicate	0.40±0.01	0.39±0.08	0.38±0.05	0.39±0.01	0.51±0.01	0.39±0.04	0.37±0.06	ND	0.62±0.15	0.34±0.02	b
2	octamethylcyclotetrasiloxane (D4)*	1.8±0.2	2.2±0.1	10.9±0.9	4.7±0.90	21.4±3.6	4.6±0.1	8.0±1.0	<LOQ	11.5±1.1	3.3±0.2	a
3	cyclosiloxane derivative (class D3, RT= 11.11)	<LOQ	<LOQ	<LOQ	<LOQ	1.5±0.2	<LOQ	<LOQ	ND	ND	1.1±0.3	a
4	bis(trimethylsilyl) diethyl orthosilicate	<LOQ	1.0±0.1	0.34±0.06	0.23±0.08	1.1±0.2	ND	ND	ND	ND	0.68±0.14	b
5	1,1,3,3,5,5,7,7,9,9-decamethylpentasiloxane (L5)	0.90±0.04	ND	0.49±0.09	ND	0.78±0.11	ND	ND	0.91±0.12	0.70±0.01	8.2±1.1	b
6	decamethylcyclopentasiloxane (D5)*	4.4±0.5	<LOQ	183.5±8.9	3.2±0.7	153.4±9.5	16.6±2.8	30.7±0.1	<LOQ	242.4±1.6	10.4±3.3	c
7	cyclosiloxane derivative (class D4, RT=13.63)	<LOQ	<LOQ	0.22±0.07	<LOQ	0.89±0.12	ND	<LOQ	ND	ND	1.01±0.19	a
8	dodecamethylcyclohexasiloxane (D6)*	149.4±13.2	15.4±0.7	546.6±59.9	263.5±18.3	465.4±24.5	231.3±7.1	55.2±4.1	<LOQ	<LOQ	167.8±1.3	d
9	Dimethylphthalate*	3.6±0.7	17.6±2.6	2.3±0.1	11.0±1.8	3.7±0.6	7.3±0.3	3.4±0.6	3.5±0.3	0.38±0.03	23.3±3.0	e
10	tetradecamethylcycloheptasiloxane (D7)	144.5±3.5	126.2±12.1	236.6±6.7	168.6±3.1	137.9±4.9	123.9±6.9	27.5±2.5	<LOQ	251.7±7.8	131.2±8.8	c
11	hexadecamethylcyclooctasiloxane (D8)	246.7±7.4	260.9±35.1	267.4±2.1	256.6±19.1	181.7±7.6	224.9±20.3	91.3±8.2	304.9±23.8	278.8±15.6	239.9±26.3	c
12	octadecamethylcyclononasiloxane (D9)	190.1±4.5	195.0±22.5	182.0±3.8	184.4±31.4	153.3±9.8	178.4±8.4	86.6±1.3	229.1±14.4	210.9±10.1	178.0±17.2	c
13	1,1,1,3,3,5,5,7,7,9,9,11,11,13,13,15,15,17,17,17-icosamethylnonasiloxane (L9)	0.65±0.02	<LOQ	<LOQ	<LOQ	<LOQ	<LOQ	<LOQ	<LOQ	2.48±0.08	0.26±0.01	b

No.	Compound	Migration concentration (mg kg^{-1})										QS
		DM15	DM25	EM18	EM20	JM10	JM20	JM24	JM25	TM17	TM18	
14	eicosamethylcyclodecasiloxane (D10)	123.2±2.4	123.2±12.2	114.3±4.9	135.9±7.1	113.1±8.5	113.4±6.4	68.1±2.2	155.3±7.3	131.5±4.1	112.4±10.2	c
15	dibutylphthalate	1.9±0.2	34.1±0.9	11.4±2.0	8.0±0.9	<LOQ	27.9±3.4	32.3±3.3	<LOQ	<LOQ	37.2±0.8	f
16	1,1,1,3,3,5,5,7,7,9,9,11,11,13,13,15,15,17,17,19,19,19-docosamethyldecasiloxane (L10)	1.3±0.1	ND	0.65±0.25	ND	0.54±0.06	ND	ND	ND	4.5±0.3	1.2±0.1	b
17	cyclosiloxane derivative (class D10, RT= 23.88)	205.9±2.5	210.8±19.6	193.8±10.9	233.1±12.6	204.7±15.6	196.6±6.5	132.1±2.4	ND	216.9±7.1	194.9±15.0	b
18	linear siloxane derivative (L10, RT= 24.44)	1.7±0.1	ND	1.4±0.3	ND	0.40±0.08	ND	ND	ND	6.3±0.5	1.4±0.1	b
19	cyclosiloxane derivative (class D10, RT= 24.80)	157.4±3.4	161.7±13.2	146.7±8.9	177.3±9.6	160.4±10.7	149.5±5.3	105.0±2.4	<LOQ	167.8±4.4	155.3±1.8	c
20	cyclosiloxane derivative (class D10, RT= 25.16)	170.4±3.9	173.3±15.2	158.0±10.0	191.4±9.5	173.8±12.1	162.1±6.2	113.9±2.7	<LOQ	180.9±4.1	167.1±1.7	c
21	linear siloxane derivative (L10, RT= 25.72)	1.4±0.1	ND	1.5±0.3	0.2±0.1	0.37±0.02	ND	0.14±0.04	<LOQ	6.7±0.6	1.1±0.1	b
22	cyclosiloxane derivative (class D10, RT= 26.36)	122.9±3.2	128.4±10.4	115.9±8.9	139.5±8.6	130.3±8.9	116.3±4.4	85.3±1.9	<LOQ	133.8±2.3	122.6±4.2	c
23	cyclosiloxane derivative (class D10, RT= 27.15)	167.6±4.1	171.9±15.5	157.2±13.4	186.9±12.6	183.9±11.1	155.8±5.4	119.4±3.8	<LOQ	182.4±4.5	164.8±5.8	c
24	linear siloxane derivative (L10, RT= 28.01)	0.54±0.06	ND	0.89±0.17	ND	ND	ND	ND	ND	4.10±0.36	0.32±0.10	b
25	cyclosiloxane derivative (class D10, RT= 28.54)	154.6±6.7	157.2±14.7	145.5±13.7	144.9±20.0	176.4±7.8	142.1±5.6	111.5±5.9	<LOQ	169.1±3.5	152.3±6.2	c
26	cyclosiloxane derivative (class D10, RT= 29.51)	126.5±5.3	131.9±10.9	119.3±9.6	116.8±12.6	147.6±7.4	113.4±4.1	90.4±4.1	<LOQ	140.2±2.2	124.4±6.3	c
27	cyclosiloxane derivative (class D10, RT= 30.45)	122.6±7.6	129.6±11.9	119.2±9.2	106.7±8.7	144.4±8.6	106.4±3.1	90.1±6.2	<LOQ	134.1±5.3	124.4±7.1	c

No.	Compound	Migration concentration (mg kg^{-1})										QS
		DM15	DM25	EM18	EM20	JM10	JM20	JM24	JM25	TM17	TM18	
28	cyclosiloxane derivative (class D10, RT= 31.49)	77.8±8.7	103.5±17.8	94.9±1.7	68.2±4.1	110.9±2.3	71.2±2.7	66.2±7.1	<LOQ	89.9±12.7	101.9±5.8	c
29	cyclosiloxane derivative (class D10, RT= 32.76)	27.3±2.6	28.5±1.4	42.9±1.4	21.9±1.4	44.4±2.9	22.8±1.4	25.1±4.7	<LOQ	25.1±1.4	47.0±3.4	c
30	cyclosiloxane derivative (class D10, RT=34.33)	5.9±0.8	12.1±0.2	8.9±1.5	4.6±0.4	10.1±1.4	4.8±0.7	5.4±1.3	<LOQ	7.3±1.1	11.8±0.7	c

<LOD: below the limit of detection; ND = Not detected; <LOQ: below the limit of quantification; QS: quantification standard used (see Table S2); *: compound with an available analytical standard; RT: retention time used to differentiate multiple signals of Dn and Ln derivatives.
